# Strategies in umbilical cord-derived mesenchymal stem cells expansion: influence of oxygen, culture medium and cell separation

**DOI:** 10.1186/1753-6561-5-S8-P88

**Published:** 2011-11-22

**Authors:** Antonina Lavrentieva, Tim Hatlapatka, Ramona Winkler, Ralf Hass, Cornelia Kasper

**Affiliations:** 1 Leibniz University Hannover, Institute of Technical Chemistry, Callinstr. 5, D-30167 Hannover; 2 Hannover Medical School, Klinik für Frauenheilkunde und Geburtshilfe, AG Biochemie und Tumorbiologie, Carl-Neuberg-Str. 1, D-30625 Hannover

## Background

Mesenchymal stem cells (MSC) from different sources attract tremendous interest in cell-based therapies for their ability to differentiate into different cell lineages. MSC are already used in clinical trials as cell therapy [1]. In comparison to other “classical” sources of MSC (e.g. bone marrow and adipose tissue), the umbilical cord (UC) matrix has great potential, since there are no ethical limitations, risks for the donor or problems with variety of donor age [2]. However, a large numbers of cells (about 10^6^ cells per 1 kg body weight) are required which may still limit the implant preparation for clinical applications. Thus, strategies for the expansion of MSC must be well defined and controlled conditions need to be developed and established for reproducible production of cells under GMP conform conditions. Conventional *in vitro* cell cultivation is carried out under ambient oxygen concentration (21% of O_2_) which is defined as “normoxic”. MSC *in vivo* usually are not exposed to such a high concentration of oxygen. In our work we studied the influence of oxygen concentration on the long-term as well as glucose and oxygen concentration on the short-term cultivation and expansion of UC-MSC.

## Materials and methods

UC-MSC were isolated from whole human umbilical cords using an explant culture approach and characterised as described earlier [3,4]. For the long-term cultivation, MSC from the same donor were isolated and subsequently cultivated in two different oxygen concentrations (5% and 21%). After isolation, cells were seeded at a density of 2000 cells/cm^2^ in 25cm^2^ cell culture flasks (Corning, Germany) and sub-cultivated every 3-4 days over 25 passages. Cell numbers were estimated at the end of each passage and cumulative population doublings were calculated for each culture conditions. MSC were cultivated in αMEM containing 1 g/l glucose (Biochrom, Germany), 10% allogenic human serum (provided by the Institute of Transfusion Medicine, Medical University Hannover, Germany) and 50 µg/ml gentamicin (PAA Laboratories GmbH). To reveal the influence of glucose concentration on the proliferation capacities of UC-MSC under different oxygen concentrations, cells were seeded in 6-well plates (Sarstedt, Germany) at a density of 700 cells/cm^2^ in αMEM (1 g/l glucose) and DMEM (4.5 g/l glucose) and cultivated over 7 days until full confluency was reached. Cell number and viability in all experiments were determined by trypan blue exclusion (n=4).

Counterflow centrifugal elutriation (CCE) was performed for the separation of the cells according to their physical size (Beckmann J6-MC with the JE-5.0 rotor; 5 ml-standard elutriation chamber, Beckman Coulter, Germany). Small-sized MSC were separated from the primary heterogeneous population. After cell separation, proliferation activity of the cells was estimated as described above.

## Results

**Figure 1:**
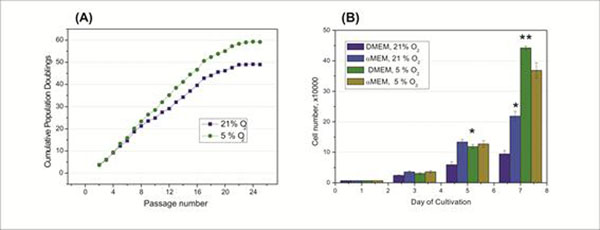
(A) Influence of different oxygen concentrations (5 % and 21%) on the long-term UC-MSC cultivation: cells were counted at the end of each passage and cumulative population doublings were calculated. (B) Influence of glucose concentration on the short-term proliferation of the MSC: cell growth in high-glucose medium is inhibited under 21% oxygen.

Long-term cultivation of UC-MSC under hypoxia revealed higher proliferation activity of the cells without changes in morphology when compared to ambient (21%) oxygen concentration (Fig 1A).

High glucose concentration inhibited cell growth under ambient oxygen concentration. Hypoxic conditions, however, significantly increased proliferation of UC-MSC both, in high- and low-glucose culture medium (Fig.1B).

## Conclusions

Application of MSC in regenerative medicine as cell suspensions or as a part of tissue engineered implant requires large amount of cells of high quality. MSC expansion under physiological conditions allows obtaining higher cell numbers in a shorter period of time. Also the glucose concentration in medium should be close to that *in vivo*. High amounts of glucose inhibit cell proliferation in ambient oxygen concentrations. In hypoxic conditions MSC proliferate better and retain their ability to differentiate. We conclude that GMP-conform cultivation of MSC with allogenic human serum under physiological oxygen concentrations as well as isolation of actively dividing cells may increase the efficiency of the cell expansion for further therapeutic applications.
